# Ferromagnetism emerged from non-ferromagnetic atomic crystals

**DOI:** 10.1038/s41467-023-39002-6

**Published:** 2023-06-29

**Authors:** Cheng Gong, Peiyao Zhang, Tenzin Norden, Quanwei Li, Zhen Guo, Apoorva Chaturvedi, Arman Najafi, Shoufeng Lan, Xiaoze Liu, Yuan Wang, Shi-Jing Gong, Hao Zeng, Hua Zhang, Athos Petrou, Xiang Zhang

**Affiliations:** 1 Nano-scale Science and Engineering Center (NSEC), 3112 Etcheverry Hall, University of California, Berkeley, CA USA; 2 Department of Electrical and Computer Engineering and Quantum Technology Center, University of Maryland, College Park, MD USA; 3 Department of Physics, University at Buffalo, State University of New York, Buffalo, NY USA; 4 Center for Programmable Materials, School of Materials Science and Engineering, Nanyang Technological University, Singapore, Singapore; 5 Engineering Research Center of Nanophotonics Advanced Instrument (Ministry of Education), Department of Physics, School of Physics and Electronic Science, East China Normal University, Shanghai, China; 6 Department of Chemistry, City University of Hong Kong, Kowloon, Hong Kong China; 7 Hong Kong Branch of National Precious Metals Material Engineering Research Center (NPMM), City University of Hong Kong, Kowloon, Hong Kong China; 8 Shenzhen Research Institute, City University of Hong Kong, Shenzhen, China; 9 Faculties of Science and Engineering, The University of Hong Kong, Hong Kong, China

**Keywords:** Magnetic properties and materials, Ferromagnetism

## Abstract

The recently emerged ferromagnetic two-dimensional (2D) materials provide unique platforms for compact spintronic devices down to the atomic-thin regime; however, the prospect is hindered by the limited number  of ferromagnetic 2D materials discovered with limited choices of magnetic properties. If 2D antiferromagnetism could be converted to 2D ferromagnetism, the range of 2D magnets and their potential applications would be significantly broadened. Here, we discovered emergent ferromagnetism by interfacing non-magnetic WS_2_ layers with the antiferromagnetic FePS_3_. The WS_2_ exhibits an order of magnitude enhanced Zeeman effect with a saturated interfacial exchange field ~38 Tesla. Given the pristine FePS_3_ is an intralayer antiferromagnet, the prominent interfacial exchange field suggests the formation of ferromagnetic FePS_3_ at interface. Furthermore, the enhanced Zeeman effect in WS_2_ is found to exhibit a strong WS_2_-thickness dependence, highlighting the layer-tailorable interfacial exchange coupling in WS_2_-FePS_3_ heterostructures, which is potentially attributed to the thickness-dependent interfacial hybridization.

## Abstract (web summary)

The isolation of graphene leads to a surge of interest in two dimensional materials, and recently, ferromagnetism has been observed in several two-dimensional materials. However, two-dimensional ferromagnetism remains rare. Here, Gong et al present an alternative approach to achieve two-dimensional ferromagnetism; combining antiferromagnetic FePS3 with non-magnetic WS2 they find a ferromagnetic state forms at the interface of these two materials.

## Introduction

Ferromagnetic materials play foundational roles in a broad range of magnetoelectric and magneto-optical devices, including magnetoresistive memories^1^, spin field effect transistors^2^, and optical isolators^3^. Distinct magnetic properties prompt a wide variety of device functionalities. For example, permanent magnets with substantial energy products enable electromechanical devices, and transparent magnets with large Verdet constants enable nonreciprocal optical devices. Recently discovered magnetic atomic crystals^4–8^ broaden the landscape of two-dimensional (2D) materials, provide ideal platforms for fundamental physics, and open unprecedented opportunities for ultracompact spintronic and magnonic devices. Combined with the incredible variety of 2D electronic and photonic materials, 2D magnets can significantly expand the atomic-thin magnetoelectric and magneto-optic functionalities. However, the fundamental hindrance to the prospect is the scarcity of 2D ferromagnets.

In stark contrast, antiferromagnetic van der Waals (vdW) crystals are much more abundant. Transition metal phosphorous trichalcogenides (MPX_3_: M = Fe, Mn, Ni, Cd, Hg; X = S, Se) are such a family with fertile magnetic configurations such as Ising antiferromagnets FePS_3_ and FePSe_3_^9–13^, Heisenberg antiferromagnets MnPS_3_ and MnPSe_3_^13,14^, and XY-type antiferromagnet NiPS_3_^14^. Most compelling promises that antiferromagnets hold stem from their attractive characteristics: immunity to the environmental magnetic field perturbation because of the alternatively opposite magnetic moments, the null stray field for eliminated crosstalk between neighboring bits, and high magnetic resonance frequency for terahertz devices (in contrast to gigahertz frequency for ferromagnets) as spin reorientation in zero-magnetization antiferromagnets does not require angular momentum transfer between the spin system and external systems (e.g., the lattice). Nevertheless, the exploration of antiferromagnets confronts the intrinsic challenge of information reading due to the net vanishing magnetization. Creating 2D ferromagnetism out of vdW antiferromagnets, if could be realized, would inherently integrate the advantages of both ferromagnets and antiferromagnets (e.g., readability of ferromagnets and ultrafast switching of antiferromagnets), leading to potential breakthroughs in high-speed low-power spintronics based on atomic crystals.

## Results

Here we discovered the emergent ferromagnetism from antiferromagnetic vdW crystal FePS_3_ based on heterostructure engineering. We chose to study the heterostructure of non-magnetic WS_2_ on the antiferromagnetic FePS_3_ substrate, out of four reasons. Firstly, antiferromagnetic FePS_3_ likely becomes a ferromagnet, for example by charge transfer doping^15,16^. Secondly, FePS_3_ is an Ising magnet with the neighboring zigzag ferromagnetic chains antiferromagnetic coupled, whose out-of-plane oriented spin magnetic moments can couple with 2D transition metal dichalcogenides efficiently^17–19^. Thirdly, WS_2_ and FePS_3_ have similar band positions and the same chalcogen species^20,21^, and therefore interfacial wavefunction may overlap efficaciously, leading to strong exchange interaction. Lastly, WS_2_ is an optically active semiconductor and can act as a sensor to the nearby time-reversal symmetry breaking events^22–27^. In this heterostructure of non-ferromagnetic vdW crystals, we discovered the interfacial ferromagnetism with maximum interfacial exchange field ~38 Tesla. Our discovery of ferromagnetism from non-ferromagnetic crystals, with a giant interfacial exchange field, opens a door to creating artificial 2D ferromagnets and developing emergent 2D antiferromagnetic spintronics and valleytronics.

**Fig. 1:**
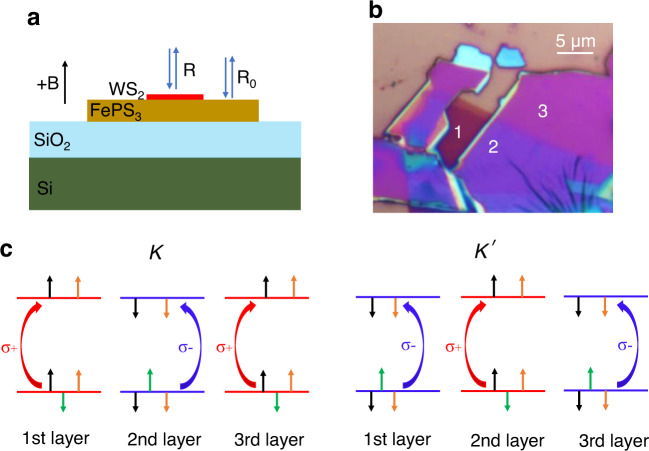
Optical image of a WS_2_-FePS_3_ heterostructure and optical selection rule of trilayer WS_2_. **a** Side-view illustration of the WS_2_-FePS_3_ heterostructure. Circularly polarized lights are incident on the WS_2_-FePS_3_ stack and a bare FePS_3_ flake, and reflected lights are collected as “R” and “R_0_”, respectively. The applied magnetic field points out-of-plane. Reflectance spectra in Fig. 2 are calculated by R/R_0_. **b** An optical image of trilayer WS_2_ on FePS_3_ on the 260-nm-SiO_2_/Si substrate. Numbers “1”, “2”, and “3” mark the regions of trilayer WS_2_ on SiO_2_/Si, trilayer WS_2_ on FePS_3_ on SiO_2_/Si, and FePS_3_ on SiO_2_/Si. **c** The layer-resolved optical selection rule for the circularly polarized light absorption for the inter-band transition at K and K′ points of trilayer WS_2_. Due to the much stronger intralayer spin orbit coupling than the interlayer coupling, the spin-valley-layer locking effect remains robust in trilayer WS_2_. Left circularly polarized light is always preferentially absorbed for the inter-band excitation of spin-up polarized electrons at Brillouin zone corners, and right circularly polarized light for spin-down polarized electrons. Black, orange, and green arrows represent spin, valley, and orbital magnetic moments, respectively. Given the same spin magnetic moments of electrons at K (or K′) points of conduction and valence band edges, the external magnetic field does not change the band gap size at K (or K′) points due to its effect on spin magnetic moments. Valley magnetic moments at K (or K′) points of conduction and valence band edges could differ only if the effective mass of electrons and holes differ. Our experimental results show this difference is small. Orbital magnetic moments at K (or K′) points of conduction and valence band edges differ, as shown by the only green arrows in valence bands. Therefore, for all three layers, an external magnetic field causes the direct-gap size of the spin-up polarized bands (green arrow up) at K points to increase and that of the spin-down polarization (green arrow down) at K′ points to decrease, or vice versa. Although the photoluminescence of the multilayer WS_2_ is quenched, optical reflectance spectra allow the probing of the direct-gap size at K or K′ points.

In this work, we synthesized bulk FePS_3_ crystals by chemical vapor transport and applied adhesive tapes to obtain freshly cleaved surfaces of FePS_3_ flakes on 260 nm SiO_2_ on Si. Monolayer and few-layer WS_2_ flakes were exfoliated on polydimethylsiloxane (PDMS). Heterostructures were prepared by direct mechanical deposition of WS_2_ on the FePS_3_-SiO_2_-Si stack, as shown in Fig. 1a, b. Consistent with the literature^9–11^, our bulk FePS_3_ is an easy-axis antiferromagnet with Néel temperature at about 120 K, as shown in Fig. S1 in the supplementary information. Given the large lattice mismatch between FePS_3_ and WS_2_, we expect the effect of the interlayer registry and twist^28^ if any would be averaged out, which is therefore not the focus of this work.

For single layer WS_2_, two degenerate but inequivalent valleys are present at K and K′ points (neighboring corners of the hexagonal Brillouin zone). Circularly polarized photons with opposite chirality can be absorbed for the inter-band excitations at K and K′ points, respectively. For multilayer WS_2_, neighboring layers have 180° relative rotation leading to the overlap of “K point of one layer” and “K′ point of the neighboring layer”, hence eliminating the valley inequivalence for the even number of layers. But in fact, transition metal dichalcogenides possess unique spin-valley-layer locking effect: spin and valley remain coupled (Fig. 1c) in each constituent layer, due to the much weaker interlayer interaction than the intralayer spin orbit coupling. For all layers, left circularly polarized photons (σ + ) are always preferentially absorbed to excite the inter-band transition between spin-up bands (red lines, Fig. 1c) at corners of Brillouin zone, and right circularly polarized photons (σ−) for spin-down bands (blue lines, Fig. 1c)^29,30^. Therefore, the absorption or reflectance difference of opposite circularly polarized photons by WS_2_ allows the detection of the nearby time-reversal-symmetry breaking phenomena.

**Fig. 2:**
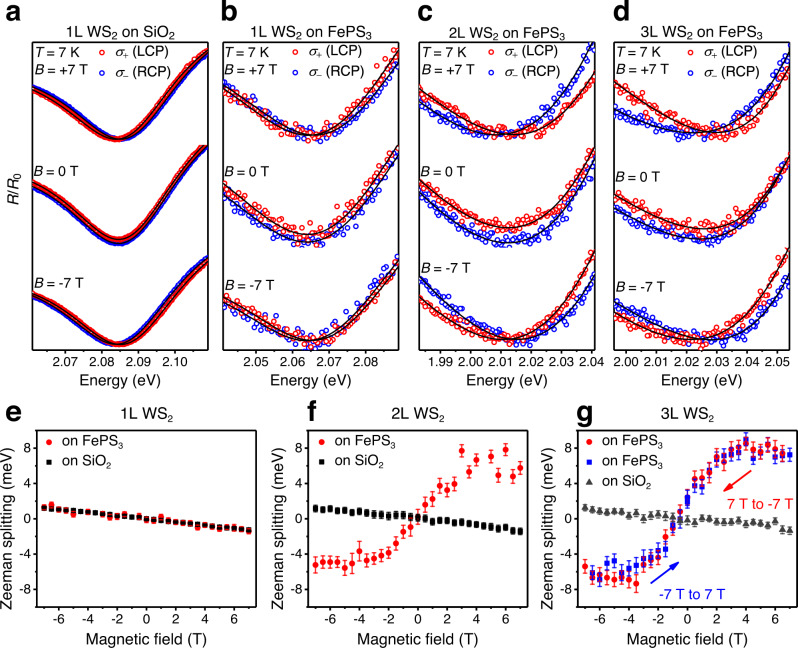
Magnetoreflectance spectra and spin splittings of monolayer, bilayer and trilayer WS_2_ on FePS_3_ and SiO_2_ at 7 K. **a**–**d** Magnetoreflectance spectra of monolayer WS_2_ on SiO_2_ **a**, and monolayer **b**, bilayer **c**, and trilayer **d** WS_2_ on thick FePS_3_. Red and blue dots represent reflectance spectra of left and right circular polarized lights. Solid lines were fitted using a complex (absorptive plus dispersive) Fano line shape to extract the absorption transition energies. Magnetoreflectance spectra of bilayer and trilayer WS_2_ on SiO_2_ are presented in Figs. S3a, b. **e**–**g** Spin splittings of monolayer **e**, bilayer **f**, and trilayer **g** WS_2_ on FePS_3_ and on SiO_2_, respectively. All different layers of WS_2_ on SiO_2_ exhibit the intrinsic Zeeman effect ~ −0.2 meV/T. Monolayer WS_2_ on FePS_3_ exhibit the intrinsic Zeeman effect as well. In stark contrast, bilayer and trilayer WS_2_ on FePS_3_ show an order of magnitude enhancements in lower magnetic field range −3.5 ~ 3.5 T, and saturate beyond  3.5 T. After saturation, the splitting drops slightly following the intrinsic Zeeman effect caused by external magnetic field. The “S”-shape magnetic field dependent spin splitting reveals the presence of interfacial ferromagnetism. The steeper slope in the low magnetic field range and the larger saturated spin splitting for the trilayer WS_2_/FePS_3_ heterostructure, compared with the bilayer WS_2_/FePS_3_ heterostructure, shows the stronger ferromagnetism. In **g**, the overall shift of the hysteresis loop to −0.5 ~ −1 T indicates a possible formation of an exchange bias between the ferromagnetic surface and the bulk of the antiferromagnetic FePS_3_ substrate. Error bars arise from the uncertainty of fitted dip positions. The dip extraction of the magnetoreflectance spectra is explained in Fig. S2 and related texts in supplementary information, and the magnetoreflectance spectra of the bilayer and trilayer WS_2_ on SiO_2_ are  shown in Fig. S3.

Figure 2a, e shows monolayer WS_2_ on SiO_2_ exhibits the Zeeman splitting at 7 K, linearly dependent on the externally applied magnetic field with a slope ~ −0.2 meV/T, which arises primarily from the different magnetic field responses of distinct *d*-orbital magnetic moments at conduction band edge (m_z_ = 0) and valence band edge (m_z_ = ±2) at K (or K’) points^22–27,30^. For monolayer WS_2_ on FePS_3_, Zeeman splitting akin to that of WS_2_ on SiO_2_ was observed, which accords with the fact that antiferromagnetic FePS_3_ has zero magnetization and thus does not enhance the Zeeman effect. Even though the antiferromagnetic FePS_3_ and diamagnetic SiO_2_ possess different magnetic susceptibility, the induced magnetic moments in both FePS_3_ and SiO_2_ are far from saturation and their dipolar effects on WS_2_ are negligible. In addition, the polarized photoluminescence (PL) spectra from monolayer WS_2_-FePS_3_ show a linear magnetic field dependence of valley Zeeman splitting (Fig. S2). The slightly larger Zeeman coefficient in PL data than in reflectance data is due to the fact that the PL herein is emitted from defect states, which usually exhibit a larger g-factor than neutral excitons^31,32^. Therefore, both PL and reflectance measurements do not show evidence of ferromagnetism in monolayer WS_2_-FePS_3_ heterostructure.

Surprisingly, still on FePS_3_, at 7 K, bilayer WS_2_ (Fig. 2c, f) exhibits a dramatically enhanced Zeeman effect. At the lower field range (e.g., −2 ~ 2 T), the spin splitting almost linearly depends on the external field with an order of magnitude larger Zeeman coefficient than the intrinsic Zeeman effect of WS_2_. Given this splitting is from reflectance spectra, the unusually large g-factors of defect states^31,32^ in monolayer transition metal dichalcogenides accessed by PL are ruled out. When the external magnetic field is larger than 3 T, the spin splitting saturates. Such “S”-shape field-dependence, together with its temperature-insensitivity (shown later)), rules out the giant-paramagnetic response in WS_2_ when Fermi level resides at a particular point between spin-orbit-coupling split conduction bands^33,34^. Rather, it is evidence of the interfacial ferromagnetism^35,36^.

To further study the effect of WS_2_ thickness, we continued on a trilayer WS_2_ on FePS_3_. Similar “S”-shape magnetic field dependence of spin splitting was observed, as shown in Fig. 2g. A steeper slope and a larger saturated spin splitting were observed in the trilayer WS_2_-FePS_3_, with respect to the bilayer WS_2_-FePS_3_. The saturated splittings for the bilayer and trilayer WS_2_ are about 5.5 meV and 7.5 meV, respectively, when the external magnetic field amounts to ~3.5 T in both cases. Under the external magnetic fields higher than 3.5 T, the magnitude of the spin splitting reduces slightly following the intrinsic Zeeman effect ~ −0.2 meV/T. The opposite sign of the enhanced Zeeman effect (due to magnetic proximity effect) with respect to the intrinsic Zeeman effect could relate to the specific interfacial magnetic coupling (e.g., antiferromagnetic interfacial proximity effect^37^ between ferromagnetic FePS_3_ surface and WS_2_), which again highlights the fundamental difference between magnetic proximity effect and Zeeman effect. The negligible opening in the hysteresis loops for both bilayer and trilayer WS_2_-FePS_3_ exhibit the small remanence which may result from many small-size domains that cannot be resolved by our optical approach.

Regarding the effect of WS_2_ thickness on the interfacial ferromagnetism, we deemed two mechanisms are possible. Firstly, the thicker WS_2_ with a narrower band gap has the conduction band edge closer to that of FePS_3_^20,21^, leading to the more effective wavefunction overlap of the two materials and their exchange interaction. Such interfacial hybridization is ubiquitous, orbital-dependent, and could effectively alter the magnetic properties^38,39^. Our density functional theory (DFT) calculation of WS_2_-FePS_3_ heterostructures confirms the orbital-dependent charge redistribution within FePS_3_ (Figs. S5 and S6) evolves with the WS_2_ thickness, indicating the interfacial hybridization sensitively depends on the WS_2_ thickness. Such interfacial hybridization likely causes band renormalization in WS_2_, leading to the anomalous WS_2_ thickness dependent reflectance dip positions (Fig. 2b–d: monolayer, 2.065 eV; bilayer, 2.015 eV; trilayer, 2.028 eV) on FePS_3_ substrate. Secondly, charge transfer between WS_2_ and FePS_3_ could partially participate in triggering the magnetic phase change of  the FePS_3_ surface from antiferromagnetism to ferromagnetism. However, our DFT calculations indicate the role of charge transfer if any would be secondary, as the amount of interfacial charge transfer is not significant enough for a magnetic phase change^40^ (see discussions in supplementary information).

The saturated exchange fields between FePS_3_ and the bilayer and trilayer WS_2_ are about 28 and 38 Tesla, respectively. Considering the measured splitting is an average result from multiple WS_2_ layers, the exchange fields between the very interfacial layer of WS_2_ and FePS_3_ can be even stronger. The giant interfacial exchange field strengths are roughly three times of that in previously studied WSe_2_/EuS, WSe_2_/CrI_3_, and graphene/EuS systems^36,41,42^, and of the same order as the exchange field strengths observed in WS_2_/EuS heterostructures^37^. The interfacial exchange interaction depends on the specific wavefunction overlap between the two materials, and becomes especially strong when the interfacial interaction is mediated by the same species (such as in WS_2_/EuS)^37^. As analyzed above, the close band positions and the same chalcogen species of WS_2_ and FePS_3_, could be important factors for the observed giant interfacial exchange field. To confirm the role of the same interfacial chalcogen species in the resultant interfacial ferromagnetism, we conducted the systematic control experiments on monolayer, bilayer and trilayer WSe_2_ on FePS_3_, and monolayer, bilayer and trilayer WS_2_ on FePSe_3_, respectively (the chalcogen species in the two constituent materials of these heterostructures are different). As shown in Fig. S7, all the studied WSe_2_/FePS_3_ and WS_2_/FePSe_3_ samples do not exhibit interfacial ferromagnetism as the WS_2_/FePS_3_ samples do. This delivers strong evidence for the role of the chalcogen mediated interfacial hybridization in the resultant interfacial ferromagnetism.

The temperature dependent study further confirms the correlation between the ferromagnetic surface and the antiferromagnetic substrate. For both bilayer and trilayer WS_2_-FePS_3_ heterostructures, the magnetic field-dependent spin splittings at 80 K agree well with that at 7 K, which implicates the application prospects above liquid nitrogen temperature. Under a fixed 5 T external field, when temperatures are elevated above 120 K, the enhanced Zeeman effect suddenly disappears and the spin splitting restores to the intrinsic values (~−1 meV at 5 T). The coincidence of the Curie temperature of the interface ferromagnetism with the Néel temperature of the antiferromagnetic FePS_3_ substrate confirms the origin of the observed ferromagnetism from the FePS_3_ surface. At 130 K, the magnetic field dependences of the spin splittings in bilayer and trilayer WS_2_ in heterostructures resemble the behaviors of their counterparts on SiO_2_ at 7 K, in both magnitude and sign, except the larger noise arising from the stronger thermal smearing of the spin-polarized bands at elevated temperatures.

**Fig. 3:**
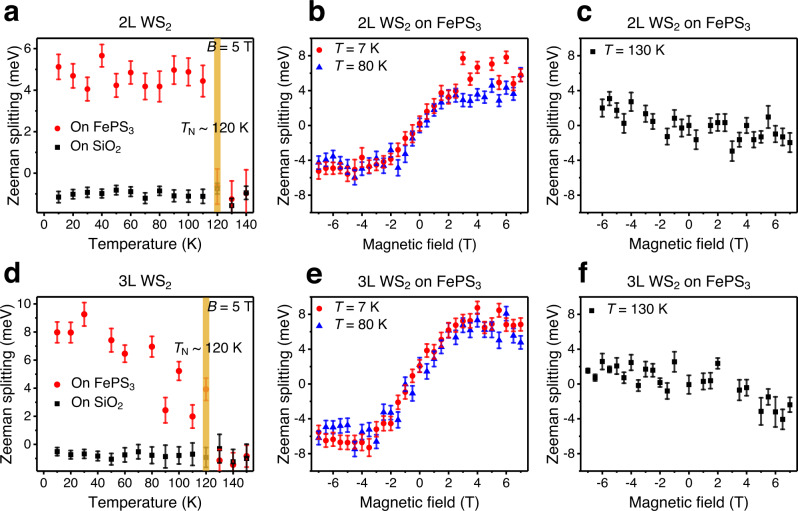
Temperature dependence of the enhanced Zeeman effects in WS_2_/FePS_3_ heterostructures. **a**, **d** Temperature dependent spin splitting for the bilayer **a** and trilayer **d** WS_2_ on FePS_3_ (versus on SiO_2_ as a reference), under 5 T magnetic field. Below 120 K, the spin splitting for WS_2_ on FePS_3_ is drastically enhanced. Above 120 K, spin splitting for WS_2_ on FePS_3_ restores to the intrinsic Zeeman splitting as on SiO_2_. **b**, **e** Magnetic field dependent spin splitting for the bilayer **b** and trilayer **e** WS_2_ on FePS_3_ at 7 K and 80 K, respectively. For both bilayer and trilayer WS_2_ on FePS_3_,  the enhanced Zeeman effects do not show a notable difference between 7 K and 80 K, implicating the application potentials above liquid nitrogen temperature. **c**, **f** Magnetic field dependent spin splitting for the bilayer **c** and trilayer **f** WS_2_ on FePS_3_ at 130 K. Above Néel temperature (~ 120 K) of the FePS_3_ substrate, the previously observed enhanced Zeeman effects disappear. The large noise in (**c** and **f**) compared with the intrinsic Zeeman effect at the lower temperature (e.g., Fig. 2f, g) is due to the thermal smearing of the spin-polarized bands at elevated temperatures. Error bars in **a**–**f** represent the uncertainty of the fitted reflectance dip positions.

The spin splittings in bilayer WS_2_-FePS_3_ almost stay constant below and decay abruptly at 120 K, but that in trilayer WS_2_-FePS_3_ decay gradually below 120 K, as shown in Fig. 3a, d. This is similar to the temperature dependent magnetization behaviors of the conventional ferromagnetic materials: under a lower magnetic field, magnetization barely changes below and abruptly drops at the transition temperature, but under a higher magnetic field, magnetization drops continuously as temperature elevates to the transition temperature (1). This behavior relates to the thermal stability of the magnetostatic energy: under a larger magnetic field, a ferromagnet has fewer domains with stronger dipolar interaction and higher magnetostatic energy; the thermal instability of the large magnetic dipolar interaction causes the gradual temperature evolution of magnetization. Our trilayer WS_2_-FePS_3_ system exhibits a stronger interfacial magnetization compared with the bilayer WS_2_-FePS_3_ system, and therefore shows a stronger temperature evolution.

In summary, the interfacial ferromagnetism is created by laying the bilayer and trilayer WS_2_ over the Ising antiferromagnet FePS_3_. Giant interfacial exchange fields enable an order of magnitude enhanced Zeeman effect in the excitonic feature of WS_2_ in WS_2_/FePS_3_ heterostructures. The high Néel temperature of antiferromagnetic FePS_3_ sustains the high temperature ferromagnetic phase at the interface. The discovery of such a ferromagnetism emerged from nonferromagnetic atomic crystals, with giant interfacial exchange fields, opens the door to activating layered antiferromagnets. The inherent integration of the antiferromagnet, strong spin-orbit coupling material and the emergent interfacial ferromagnet in this new heterostructure platform opens up new possibilities to harness emergent ferromagnetism for spintronic, valleytronic, and optoelectronic devices.

## Methods

### Growth of bulk FePS_3_ crystal

For the single crystal chemical vapor transport (CVT) growth, the stoichiometric amounts of iron powder (Aldrich, >99%), red phosphorus lump (Alfa Aesar, Puratronic®, 99.999 + % (metal basis)), and sulfur pieces (Alfa Aesar, Puratronic®, 99.9995% (metal basis)) were used. These chemicals were used as-received without further purification. The materials were sealed in an evacuated quartz tube (ampoule) with an inner pressure in the range of 10^−5 ^Torr to 10^−6 ^Torr. In addition to the elemental constituents (Fe, P, and S), a small amount (2 mg/cc) of iodine spheres were added inside the ampoule to act as the transport agent. Sealed ampoules were then subjected to a two-zone horizontal tube furnace.

Initially, the source zone was kept at 650 °C and the growth zone at 750 °C for 48 h. This was done to allow the constituents to react completely as well as prevent the back transport or formation of undesired additional phases. After this duration, the temperature of the source zone was gradually increased to 750 °C and that of the growth zone was lowered to 700 °C. This arrangement continued for the next 120 h. Further, the temperatures of both the zones were lowered down, and the plate-like crystals were subsequently obtained from the ampoule for further characterizations and measurements.

### WS_2_-FePS_3_ heterostructure assembling

The WS_2_ bulk crystals and polydimethylsiloxane (PDMS) films were  purchased from HQ Graphene and Gel-Pak, respectively. After the few-layer WS_2_ was exfoliated on PDMS films, the WS_2_-PDMS-glass slide stack was mounted to a translation stage, and moved under an optical microscope to be matched onto the exfoliated FePS_3_ flake on the 260 nm SiO_2_/Si chip. After lifting up the glass slide, the PDMS film was lifted up leaving the few-layer WS_2_ on FePS_3_ on SiO_2_/Si. Overnight annealing of the samples in high vacuum (<10^−8^ torr) at 150 °C was done before loading the samples to the cryostat for the systematic magnetoreflectance study.

### Magnetoreflectance measurements

The samples were placed on the cold finger of a continuous-flow optical cryostat operated in the 5–300 K temperature range. The cryostat was mounted on a three-axis translator with a spatial resolution of 10 µm in each direction. The cryostat tail was positioned inside the room temperature bore of a 7 T superconducting magnet. A collimated white-light beam was used for the reflectivity work. The incident light was focused on the sample using a microscope objective with a working distance of 34 mm. The incident beam was polarized either as left circular polarization (σ_+_) or right circular polarization (σ_−_) using a Babinet-Soleil compensator. The objective collected the reflected beam from the sample in the Faraday geometry and the light was focused onto the entrance slit of a single monochromator that uses a cooled charge-coupled device detector array.

### Density functional theory (DFT) calculations

First-principles calculations based on the DFT were performed by using the projector augmented wave (PAW) method implemented in Vienna Ab-initio Simulation Package (VASP)^43^. Generalized gradient approximation (GGA) in the scheme of Perdew-Burke-Ernzerhof (PBE) was used to treat the exchange-correlation potential^44^. The structures were relaxed until the Hellmann-Feynman forces on each atom are less than 0.01 eV/Å. The thickness of the vacuum region along the z axis is 15 Å. The van der Waals correction (vdW-DF) was adopted to optimize the lattice structures and bond lengths. For the Hubbard-U term^45^, Dudarev’s approach was used to treat localized *d* orbitals in Fe, using the effective U parameter of U_eff_ = 2 eV. The energy cutoff of 500 eV for the plane wave expansion and a 7 × 7 × 1 k-point grid were used for the self-consistent calculations, and the total energy was  converged within 1 × 10^−5^ eV. The in-plane lattice constant for FePS_3_ 2 × 2 × 1 supercell is 11.894 Å, and the in-plane lattice parameter for WS_2_ 4 × 4 × 1 supercell is 12.732 Å, which leads to a mismatch of ~6.6% between the FePS_3_ and WS_2_ supercell.

## Supplementary information

Supplementary Information

## Data Availability

The authors declare that the data supporting the findings of this study are available within the paper and its supplementary information files. Additional information is available from the corresponding author upon reasonable request.

## Code availability

The data analysis computer codes used in this study are available from the corresponding author upon reasonable request.
