# Supplementary material for: Ferromagnetism emerged from non-ferromagnetic atomic crystals
Source: Nat Commun. 2023 Jun 29;14:3839. doi: 10.1038/s41467-023-39002-6 (PMC10307804; doi:10.1038/s41467-023-39002-6)
Supplement: Supplementary file 1 — Supplementary Information [file 41467_2023_39002_MOESM1_ESM.pdf]

Supplementary Information for

# Ferromagnetism emerged from non-ferromagnetic atomic crystals

Cheng Gong,[1,2,&] Peiyao Zhang,[1,3,&] Tenzin Norden,[3] Quanwei Li,[1] Zhen Guo,[1] Apoorva Chaturvedi,[4] Arman Najafi,[3] Shoufeng Lan,[1] Xiaoze Liu,[1] Yuan Wang,[1] Shi-Jing Gong,[5] Hao Zeng,[3] Hua Zhang,[6,7,8] Athos Petrou,[3] Xiang Zhang[1,9,*]

[1]Nano-scale Science and Engineering Center (NSEC), 3112 Etcheverry Hall, University of California, Berkeley, California 94720, USA
[2]Department of Electrical and Computer Engineering and Quantum Technology Center, University of Maryland, College Park, Maryland 20742, USA
[3]Department of Physics, University at Buffalo, State University of New York, Buffalo, New York 14260, USA.
[4]Center for Programmable Materials, School of Materials Science and Engineering, Nanyang Technological University, Singapore, Singapore
[5]Engineering Research Center of Nanophotonics Advanced Instrument (Ministry of Education), Department of Physics, School of Physics and Electronic Science, East China Normal University, Shanghai 200241, China
[6]Department of Chemistry, City University of Hong Kong, Kowloon, Hong Kong, China
[7]Hong Kong Branch of National Precious Metals Material Engineering Research Center (NPMM), City University of Hong Kong, Kowloon, Hong Kong, China
[8]Shenzhen Research Institute, City University of Hong Kong, Shenzhen 518057, China
[9]Faculties of Science and Engineering, The University of Hong Kong, Hong Kong, China.

[&]These authors contributed equally.
[*]Email: xiang@berkeley.edu

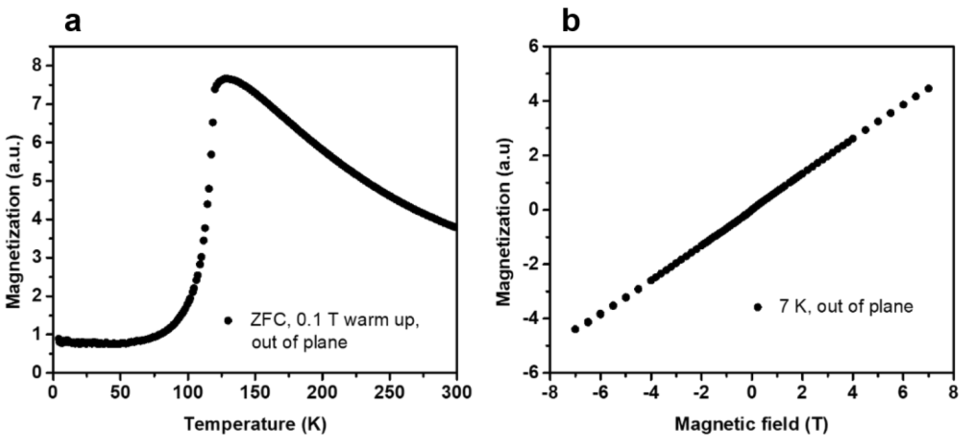


**Fig. S1 | Magnetization characterization of a bulk $FePS_3$ crystal. a**, Temperature dependent out of plane magnetization. The sample is a bulk $FePS_3$ crystal. It was cooled down under zero field, and the measurement was done during warming up process under 0.1 T. Temperature dependent magnetization clearly shows the Néel temperature at ~120 K. **b**, Magnetic field dependent magnetization. The same bulk $FePS_3$ crystal in (**a**) was measured at 7 K. The magnetization of the antiferromagnet shows a clear linear dependence on the applied magnetic field in all the range.

**Polarization-resolved photoluminescence (PL) measurements.** Systematic PL measurements are performed on monolayer $WS_2$-$FePS_3$ heterostructure in a magnetic field perpendicular to the sample plane. To extract the splitting between the K and K′ valley, the PL is excited by linearly polarized 532 nm laser and analyzed in a single helicity of light (σ+/σ-). Fig.S2, A, B and C, show PL spectra for monolayer $WS_2$-$FePS_3$ at selected values of the magnetic field *B*. The PL peak at ~2.0 eV is attributed to localized exciton (1, 2), whereas the reflectance feature at ~2.07 eV is from monolayer $WS_2$ neutral exciton as shown in Fig. 2b. At zero field, the PL spectrum from K valley (σ+, red) is identical to that from K′ valley (σ-, blue), which indicates zero energy splitting of the two valleys, as expected from time-reversal symmetry. At a high field, there is a clear splitting between the σ+ and σ- PL components. The σ+ spectrum shifts to lower (higher) energy while the σ- spectrum shifts to higher (lower) energy with an applied 7 T (-7 T) magnetic field. The Zeeman splitting is defined as $E(\sigma+) - E(\sigma-)$ and is summarized in Fig. S2d as function of magnetic field at 7 K. The Zeeman splitting of localized exciton is linearly dependent on applied magnetic field with a slope ~0.6 meV per Tesla, three times the slope for monolayer $WS_2$ neutral exciton feature extracted from reflectance spectra as shown in Fig. 2d. The mechanism of

large g-factor from localized exciton remains to be understood, as already reported in Refs. 3 and 4. The linear dependence on magnetic field shows no enhanced Zeeman effect from antiferromagnetic $FePS_3$ substrate.

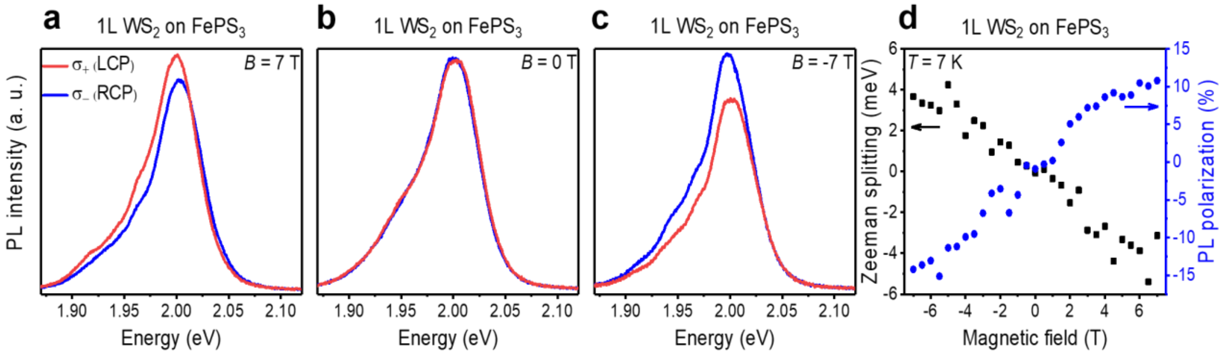


**Fig. S2 | Polarization-resolved photoluminescence spectra and spin splittings of monolayer $WS_2$ on $FePS_3$. a**,**b**,**c**, Polarization-resolved photoluminescence spectra of monolayer $WS_2$ on $FePS_3$ at selected magnetic fields: 7 T (**a**), 0 T (**b**), and -7 T (**c**). Red and blue curves represent PL analyzed in its left (σ+) and right (σ-) circular polarized components. **d**, Spin splitting and PL polarization for the localized excitons from monolayer $WS_2$ on $FePS_3$. The linear dependence of Zeeman splitting on magnetic field with a slightly larger slope than neutral excitons is consistent with other reports elsewhere for defects states, indicating no enhanced Zeeman effect from antiferromagnetic $FePS_3$ substrate.

**Reflectance dip extraction algorithm.** As shown in Fig. S3, the reflectance spectrum for the exciton was calculated using on flake/off flake reflectance ratio and fitted using a combination of absorptive and dispersive lineshapes in addition to a linear background. The dip energy was extracted using the local minimum of the fitted curve.

The fitting function (Breit-Wigner-Fano function):

$$f(x) = A\frac{(q\sigma/2 + x - \mu)^2}{(\sigma/2)^2 + (x-\mu)^2}$$

Where $A$ is amplitude, $q$ is Fano parameter which represents the ratio of resonant scattering to the background scattering, $\sigma$ is width of the lineshape, $\mu$ is center of the lineshape.

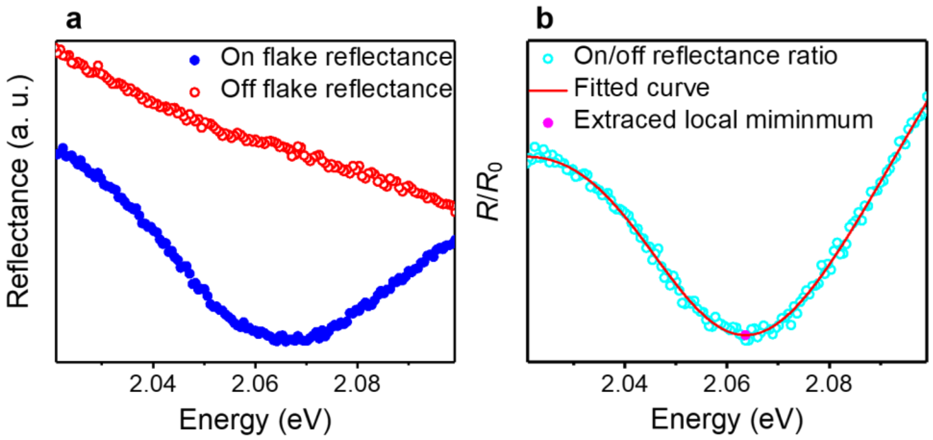


**Fig. S3 | An example of background removal and dip energy extraction. a**, Blue circles/red open circles show the on flake/off flake reflectance signal from a $WS_2$ sample. **b**, The cyan open circles indicate the on/off reflectance ratio. The fitted curve is shown in the red line and extracted dip position (local minimum) is represented as the magenta dot.

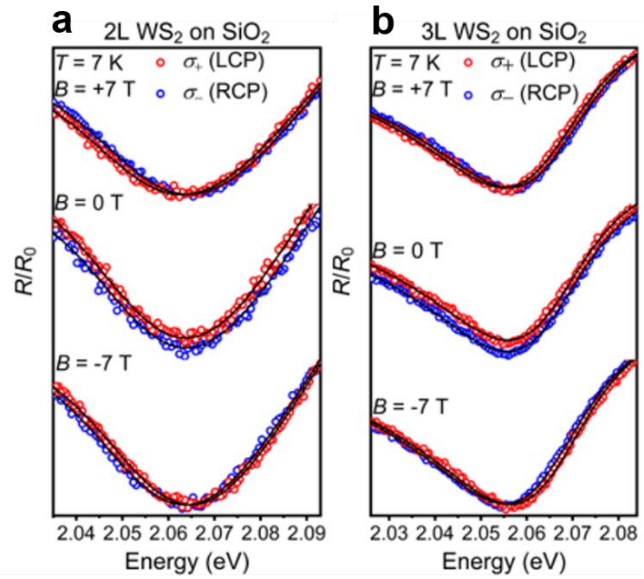


**Fig. S4 | Magnetoreflectance spectra of (a) 2L- and (b) 3L-$WS_2$ on $SiO_2$ at 7 K**. Red and blue dots represent reflectance spectra of left and right circularly polarized photons. Solid lines were fitted curves using a complex (absorptive plus dispersive) Fano line shape. Magnetoreflectance spectra of 1L-$WS_2$ on $SiO_2$ is presented in Fig. 2.

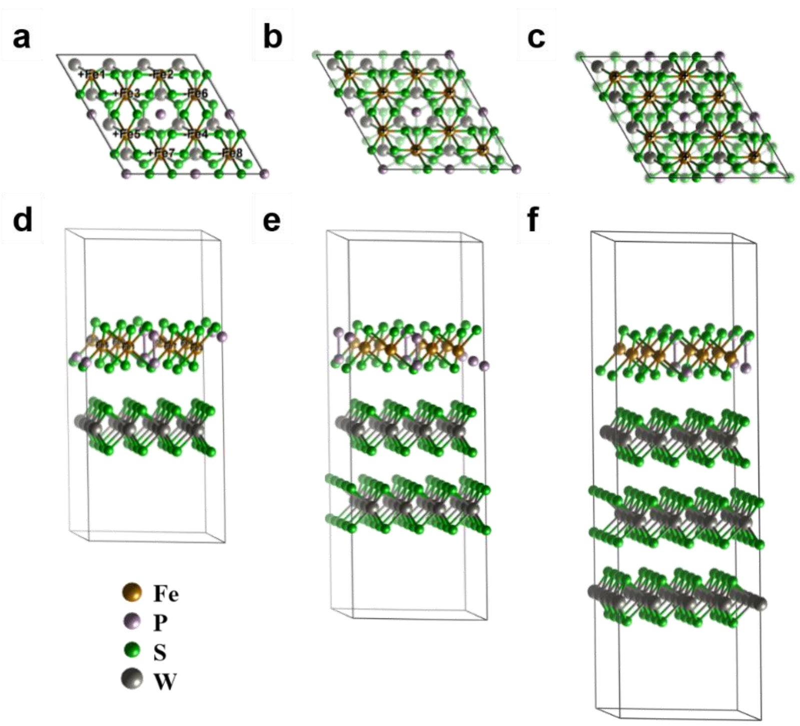


**Fig. S5 | Crystal structures of 1L-, 2L-, and 3L-$WS_2$/$FePS_3$. a**,**b**,**c**, top views. **d**,**e**,**f**, side views. (**a**, **d**), (**b**, **e**), and (**c**, **f**) are for 1L-, 2L-, and 3L-$WS_2$/$FePS_3$, respectively.

Fig. S5 shows the crystal structures of 1L-, 2L-, and 3L-$WS_2$/$FePS_3$. In Fig. S5a, the labels with '+' and '-' on Fe atoms represent the antiferromagnetic order of Fe atoms in 1L-$WS_2$/$FePS_3$. In figs. S5(b, c), for 2L- and 3L-$WS_2$/$FePS_3$, all the Fe atoms are labeled as '+' to represent ferromagnetic order.

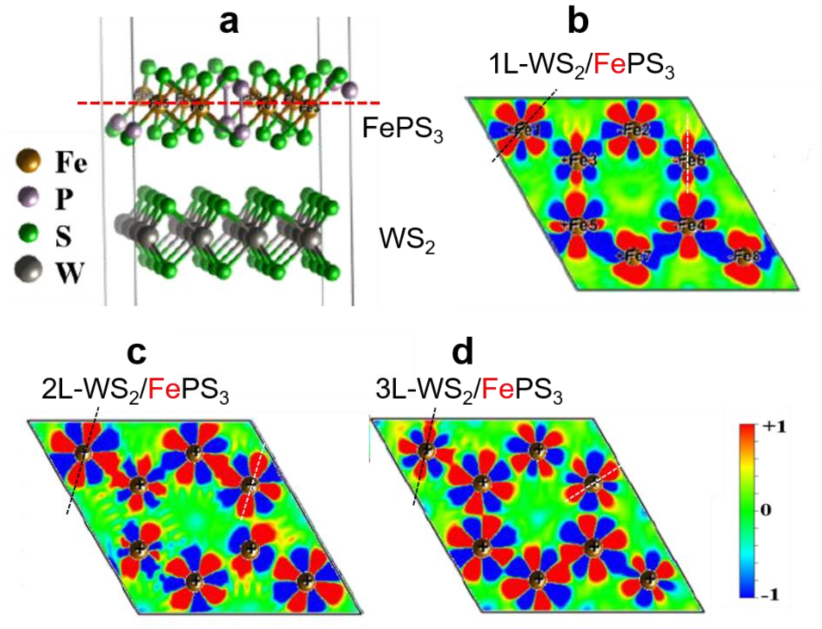


**Fig. S6 | Atomic structure (a) of interfacial layers of nL-$WS_2$/$FePS_3$ heterostructures, and the charge density difference plots (b, c, d) at the interfacial Fe layer (indicated by the red dashed line in (a)) of nL-$WS_2$/$FePS_3$ heterostructures (n = 1, 2, and 3, respectively).** Blue and red colors in (**b, c, d**) indicate the charge depletion and accumulation, and are normalized. The charge density difference is defined as $\Delta\rho = \rho_{(FePS_3\text{-}WS_2)} - \rho_{(FePS_3)} - \rho_{(WS_2)}$, where $\rho_{(FePS_3\text{-}WS_2)}$ is the charge density of the heterostructure, and $\rho_{(FePS_3)}$ and $\rho_{(WS_2)}$ are charge densities of $FePS_3$ only and $WS_2$ only, respectively. Thus, the plots here mean: while $WS_2$ comes to contact $FePS_3$, the electrons in $FePS_3$ surface layer redistribute (i.e., some orbitals lose electrons, whereas some orbitals gain electrons). Of particular interest is, if we zoom in the details in (**b, c, d**) and look at Fe1 atoms (black dashed lines serve as eye guide), the charge redistribution profiles differ among the three heterostructures. Likewise, if we zoom in the details on Fe6 atoms (white dashed lines serve as eye guide), the charge redistribution profiles also evolve among the three heterostructures. This shows that the interface wave function overlaps differently in the three heterostructures, leading to the different inter-orbital charge redistribution within $FePS_3$. Such inter-orbital charge redistribution will affect the resultant magnetic properties of $FePS_3$.

Given exchange interaction in magnetic materials is a Columbic interaction in nature, the magnetic properties of the antiferromagnets can be synergistically affected by multiple factors while interfacing with other materials (e.g., modified dielectric environment, unintentional strain,

charge transfer, spin orbit coupling proximity, etc.), as summarized in Ref. 5. Our DFT calculations based on the heterostructures consisting of $FePS_3$ and 1L-, 2L-, and 3L-$WS_2$, respectively, show the amounts of electron transfer from $FePS_3$ to $WS_2$ are ~$3\times10^{12}$ per $cm^2$ in all cases. That is, $FePS_3$ is hole doped by ~$3\times10^{12}$ per $cm^2$ by $WS_2$. If charge doping is the only driver for $FePS_3$ magnetic phase change, the hole doping should surpass $2\times10^{14}$ per $cm^2$ (6) to trigger the magnetic phase transition. This is evidence that charge transfer does not play a major role in our observed interfacial ferromagnetism. But given many factors should entangle at interface and the role of charge transfer cannot be completely excluded.

Evidence of interfacial hybridization can be seen in charge redistribution analysis. We plotted the calculated charge redistribution of heterostructures at DFT level, as shown in Fig. S6. The red and blue colors map out the regions on $FePS_3$ side where electrons are gained and lost, respectively, when $WS_2$ comes to contact $FePS_3$. Although the net interfacial charge transfer is that $FePS_3$ loses electrons to $WS_2$, $FePS_3$ does not lose electrons everywhere, and actually some orbitals of $FePS_3$ lose electrons (blue color) and some gain electrons (red color). This means, regardless of the overall interfacial charge transfer across the two dissimilar materials, there are always charge redistribution within $FePS_3$. Such a charge redistribution is driven by the interfacial wavefunction overlap, and exists in all heterosystems universally (e.g., Xe on Cu (7), graphene-metal complex (8), and $MoS_2$-metal complex (9)). As the layer number of $WS_2$ increases from 1L, 2L, to 3L, the orbital profiles of the charge redistribution in the interfacial $FePS_3$ layer evolve, as seen from the patterns in Fig. S6 (red and white dashed lines serve as eye guides for different Fe atoms). The redistributed electrons among different orbitals can effectively affect the exchange interaction and spin-orbit coupling, changing the magnetic properties of $FePS_3$.

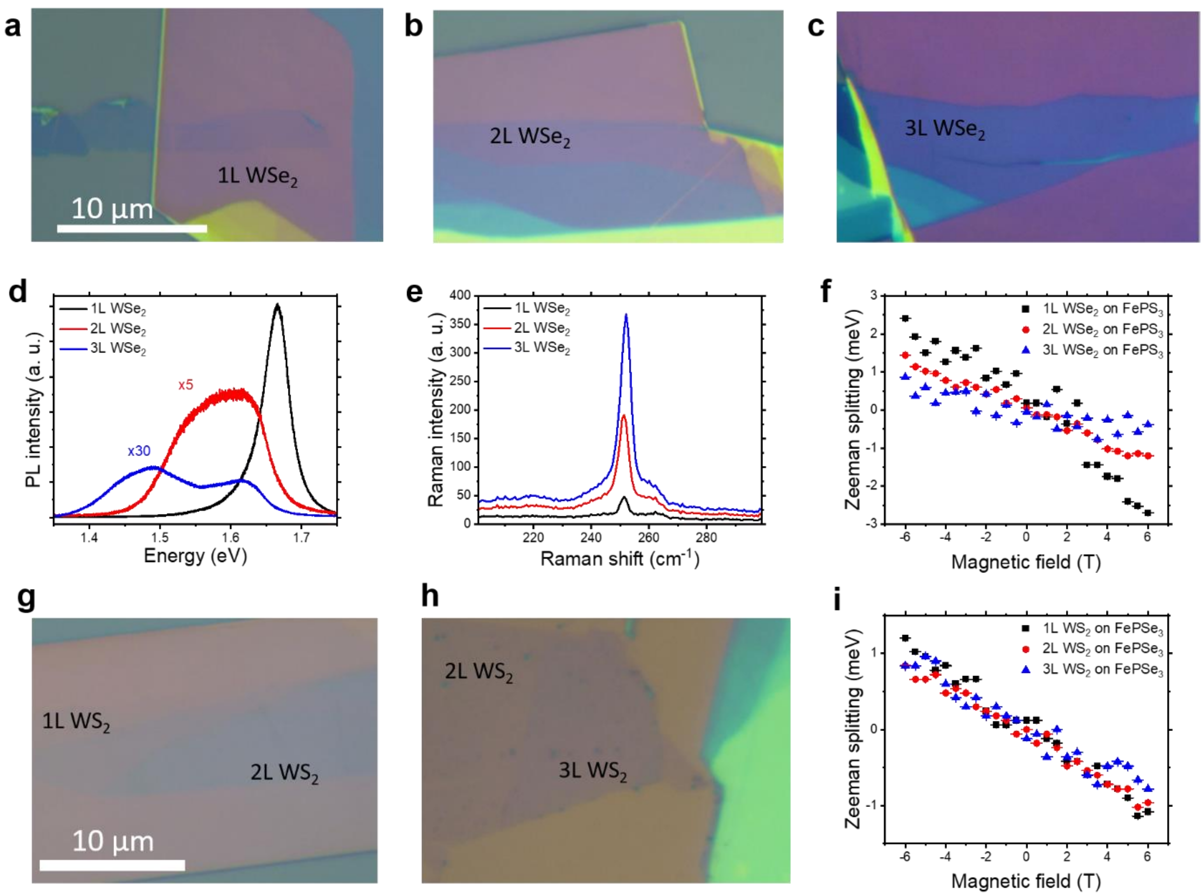


**Fig. S7 | Studies of nL-$WSe_2$/$FePS_3$ and nL-$WS_2$/$FePSe_3$ heterostructures (n = 1, 2, and 3, respectively).** Optical images of 1L-(**a**), 2L-(**b**), and 3L(**c**)-$WSe_2$/$FePS_3$, respectively. PL spectra of 1L-, 2L-, and 3L-$WSe_2$ on PDMS substrates (**d**). Raman spectra of 1L-, 2L-, and 3L-$WSe_2$ on PDMS substrates (**e**). Summarized magnetic-field-dependent Zeeman splitting of 1L-, 2L-, and 3L-$WSe_2$/$FePS_3$ (**f**). Optical images of 1L-, 2L-, and 3L-$WS_2$/$FePSe_3$ (**g**, **h**). Summarized magnetic-field-dependent Zeeman splitting of 1L-, 2L-, and 3L-$WS_2$/$FePSe_3$ (**i**). Note that both (**g**) and (**h**) show 2L-$WS_2$/$FePSe_3$ samples, and the result of the 2L-$WS_2$/$FePSe_3$ sample in (**i**) is from the sample in (**g**). The absence of interfacial ferromagnetism in all the studied $WSe_2$/$FePS_3$ and $WS_2$/$FePSe_3$ samples delivers strong evidence of the role of interfacial hybridization (mediated by the same interfacial chalcogen species) in the resultant interfacial ferromagnetism in our $WS_2$/$FePS_3$ samples.

**References:**


1. G. Plechinger *et al*., *Nat. Commun.* **7**, 12715 (2016).
2. G. Plechinger *et al*., *Phys. Status Solidi (RRL)-Rapid Res. Lett.* **9**, 457-461 (2015).
3. A. Srivastava *et al.*, *Nat. Nanotechnol.* **10**, 491-496 (2015).
4. M. Koperski *et al.*, *Nat. Nanotechnol.* **10**, 503-506 (2015).
5. C. Gong, X. Zhang, *Science* **363**, eaav4450 (2019).
6. B. L. Chittari *et al.*, *Phys. Rev. B* **94**, 184428 (2016).
7. P. S. Bagus, V. Staemmler, C. Wöll, *Phys. Rev. Lett.* **89**, 096104 (2002).
8. C. Gong *et al.*, *J. Appl. Phys.* **108**, 123711 (2010).
9. C. Gong, L. Colombo, R. M. Wallace, K. Cho, *Nano Lett.* **14**, 1714-1720 (2014).
